# Elective egg freezing and its underlying socio-demography: a binational analysis with global implications

**DOI:** 10.1186/s12958-018-0389-z

**Published:** 2018-07-23

**Authors:** M. C. Inhorn, D. Birenbaum-Carmeli, J. Birger, L. M. Westphal, J. Doyle, N. Gleicher, D. Meirow, M. Dirnfeld, D. Seidman, A. Kahane, P. Patrizio

**Affiliations:** 1 Department of Anthropology, Yale University, 10 Sachem Street, New Haven, CT 06511 USA; 2 Department of Nursing, University of Haifa, 3498838 Haifa, Israel; 3 Larchmont, USA; 4 Stanford Fertility and Reproductive Medicine Center, Stanford University, 1195 W. Fremont Ave, Sunnyvale, CA 94087 USA; 5 Shady Grove Fertility, 9600 Blackwell Road, Rockville, MD 20850 USA; 6 Center for Human Reproduction, 21 E. 69th Street, New York, NY 10021 USA; 7 Department of Obstetrics and Gynecology, Sheba Medical Center, IVF and Fertility Unit, 1 Emek Ha’ella St, 52621 Ramat Gan, Israel; 8 Division Reproductive Endocrinology-IVF, Department of Obstetrics & Gynecology, Carmel Medical Center, Ruth & Bruce Faculty of Medicine, Technion, 3436212 Haifa, Israel; 9 Assuta Medical Center, 13 Eliezer Mazal, 75653 Rishoon Lezion, Israel; 10 Yale Fertility Center, Yale University, 150 Sargent Drive, New Haven, CT 06511 USA

**Keywords:** Socio-demography, Reproductive epidemiology, Oocyte cryopreservation, Fertility preservation, Single women, Men as partners, Gender, Education, United States, Israel

## Abstract

**Background:**

What are the underlying socio-demographic factors that lead healthy women to preserve their fertility through elective egg freezing (EEF)? Many recent reviews suggest that women are intentionally postponing fertility through EEF to pursue careers and achieve reproductive autonomy. However, emerging empirical evidence suggests that women may be resorting to EEF for other reasons, primarily the lack of a partner with whom to pursue childbearing. The aim of this study is thus to understand what socio-demographic factors may underlie women’s use of EEF.

**Methods:**

A binational qualitative study was conducted from June 2014 to August 2016 to assess the socio-demographic characteristics and life circumstances of 150 healthy women who had undertaken at least one cycle of elective egg freezing (EEF) in the United States and Israel, two countries where EEF has been offered in IVF clinics over the past 7–8 years. One hundred fourteen American women who completed EEF were recruited from 4 IVF clinics in the US (2 academic, 2 private) and 36 women from 3 IVF clinics in Israel (1 academic, 2 private). In-depth, audio-recorded interviews lasting from 0.5 to 2 h were undertaken and later transcribed verbatim for qualitative data analysis.

**Results:**

Women in both countries were educated professionals (100%), and 85% undertook EEF because they lacked a partner. This “lack of a partner” problem was reflected in women’s own assessments of why they were single in their late 30s, despite their desires for marriage and childbearing. Women themselves assessed partnership problems from four perspectives: 1) women’s higher expectations; 2) men’s lower commitments; 3) skewed gender demography; and 4) self-blame.

**Discussion:**

The “lack of a partner” problem reflects growing, but little discussed international socio-demographic disparities in educational achievement. University-educated women now significantly outnumber university-educated men in the US, Israel, and nearly 75 other societies around the globe, according to World Bank data. Thus, educated women increasingly face a deficit of educated men with whom to pursue childbearing.

**Conclusion:**

Among healthy women, EEF is a technological concession to gender-based socio-demographic disparities, which leave many highly educated women without partners during their prime childbearing years. This information is important for reproductive specialists who counsel single EEF patients, and for future research on EEF in diverse national settings.

## Background

During the second decade of the new millennium, oocyte cryopreservation via vitrification has gained increasing international acceptance as a method of fertility preservation, not only for women facing fertility-threatening medical conditions [1, 2], but also for healthy women who hope to preserve their future reproductive potential [3, 4]. In the growing literature on the non-medical uses of oocyte cyropreservation, the terms “social egg freezing,” “nonmedical egg freezing,” “elective oocyte cryopreservation,” “elective fertility preservation,” and “oocyte banking for anticipated gamete exhaustion” have all been forwarded [5, 6]. Here, we suggest that “elective egg freezing” (EEF) be added to the glossary of accepted terms [7], because it may most closely mirror women’s preferred usage, per the study results described below.

Most recent reviews of oocyte cryopreservation suggest that women are undertaking EEF primarily to “delay,” “defer,” or “postpone” their fertility for educational and career purposes, thereby achieving reproductive autonomy [8], and forestalling age-related fertility decline [9–12]. However, it is unclear from these recent overviews whether postponement of fertility through EEF is intentional and planned, and whether the achievement of career advancement or reproductive autonomy are women’s primary EEF goals.

Emerging survey data among women who have completed EEF in the United States [13, 14], Belgium [15], and Australia [16, 17] paint a somewhat different picture. In three of these reports [14–16], women specifically listed “lack of a partner” as their primary reason for undergoing EEF. In the Australian study in particular, women were contacted by mail up to 15 years after completing EEF, but 90% of women had yet to use their stored oocytes. Most reported that they were still hoping to find a partner, thereby avoiding single parenthood [16]. In one of the US studies undertaken in the San Francisco Bay Area, women who had undergone EEF on average 2 years before completing an anonymous survey reported significant anxiety, depression, loneliness, and hopelessness about their reproductive futures in the absence of current male partners [13]. One in six women also experienced regret for having undertaken EEF, for reasons that remain unclear in the study.

To date, only one, small-scale, interview-based study has explored the motivations of Euro-American women who undertook EEF, including 23 in the UK [18], as well as 7 in the US, and 1 in Norway [19–21]. All of these women were heterosexual, educated (68% with postgraduate degrees or other professional qualifications), and most were in professional or managerial roles (74%). Although all of these women were hoping to be in committed relationships, 84% were single when they undertook EEF, generally toward the end of their peak reproductive life span (average age: 37).

As noted in these reports, more empirical research is clearly needed to assess the socio-demographic profiles, specific life circumstances, and reproductive outcomes of women who have undertaken EEF [16, 18]. In particular, qualitative research based on in-depth interviews with women themselves is needed to understand women’s reasons for undertaking EEF, including whether it is being used primarily for planned fertility postponement (i.e., in achieving educational or career goals during the 20s or early 30s), or whether EEF is being used primarily for fertility preservation in the absence of a committed reproductive partner (i.e., in the mid- to late-30s and early 40s).

## Methods

To answer this question, we conducted a large-scale, interview-based, qualitative study to assess women’s motivations for EEF. The study was designed to be binational and comparative across two countries, the United States and Israel, where clinical use of oocyte vitrification occurred early following removal of the experimental label in January 2011 in Israel and October 2012 in the US. American and Israeli women who had completed at least one cycle of EEF were invited to participate in in-depth, semi-structured, qualitative interviews regarding their EEF motivations and experiences. The theoretical framework of the study was thus largely person-centered and experiential [22]. However, detailed and relevant socio-demographic data were also collected from the women during interviews. Thus, socio-demographic profiles of study participants could be constructed, and EEF variables (e.g., education, career, financial stability, partnership status) and outcomes (e.g., number of cycles, egg stored, eggs used) assessed.

Between June 2014 and August 2016, 150 women volunteered to participate for this study—114 in the US and 36 in Israel. Women were recruited from 7 IVF clinics, 4 in the US (2 academic, 2 private) and 3 in Israel (1 academic, 2 private). In the US, women received study flyers by email or during clinic appointments. In Israel, women were invited by clinic staff to participate. Those women interested in volunteering for the study were then contacted by the first and second authors, both of whom are medical anthropologists with extensive experience in interviewing IVF clinic patients.

Women in both countries signed written informed consent forms, agreeing to participate in a confidential, audio-recorded interview at a time and location of their choosing. American women were interviewed by the first author in English, while Israeli women were interviewed by the second author in Hebrew. Because the American women were recruited from IVF clinics on both the East (e.g., New Haven, New York, Baltimore and Washington, DC) and West coasts (e.g., San Francisco Bay Area and Silicon Valley), approximately half of the interviews were carried out in person or by skype, and the rest by phone. In Israel, all but three interviews were conducted in person, primarily in Tel Aviv and Haifa. Interviews generally lasted 1 h, although they ranged in length from 0.5 to 2 h.

An identical interview schedule (which was also translated into Hebrew) was used to conduct interviews in both countries. Interviews were semi-structured, with both closed- and open-ended questions. All women were asked a brief series of socio-demographic questions (i.e., age, birthplace, current residence, education, employment, relationship status, ethnicity, religion), as well as reproductive history questions (i.e., menarche, contraception, reproductive health).

Open-ended questions focused on ten major themes: 1) knowledge and awareness of EEF; 2) motivations to undertake EEF; 3) EEF support systems in place; 4) EEF financing; 5) physical and emotional responses to the EEF process; 6) perceptions of EEF outcomes; 7) egg storage and future disposition; 8) retrospective reflections following EEF completion; 9) future hopes and plans for motherhood; and 10) final EEF thoughts and recommendations. The second theme regarding women’s motivations to undertake EEF is of particular interest in this paper, given the lack of consensus in the existing literature about why otherwise healthy women are pursuing EEF.

In this study, all interviews were audio-recorded and transcribed verbatim, with Hebrew interviews then translated into English by a professional translator. Detailed case summaries were then written by the two medical anthropologist interviewers after each transcription was completed. These summaries were shared and reviewed prior to qualitative data analysis, in order to agree upon a common coding scheme. Transcriptions and case summaries were then uploaded into Dedoose, a software program designed for qualitative data analysis. Dedoose allowed for key word searching and the identification of common themes and patterns emerging from the extensive interview data. Socio-demographic information collected during the interviews was also entered into Excel files for descriptive statistical analysis. The research protocol and data management plan were approved by academic Institutional Review Boards and by the ethics committees of all the collaborating IVF clinic sites.

## Results

The socio-demographic characteristics of the 150 women in the study, as well as their motivations for undertaking EEF, are supported by qualitative findings, in which women themselves provided nuanced insights on their life circumstances and motivations for EEF.

### Socio-demographic findings

In-depth interviews revealed striking similarities between American and Israeli women who had completed EEF. As shown in Table 1, about three-quarters (73%) of the women in both countries froze their eggs in their late 30s (ages 35–39), with the remainder in their early 30s (17%) or early 40s (9%). The average age for EEF in the US was 36.6, and 36.2 in Israel. Only one woman in the study froze her eggs before age 30 (an American woman who was 29).

**Table 1:** Elective Egg Freezing in the US and Israel: Characteristics of Study Participants and Their EEF Cycles

Characteristics	United States		Israel		Total	
	*n*	%	*n*	%	*n*	%
Age at EEF						
25–29	1	< 1	0	0	1	1
30–34	19	17	7	19	26	17
35–39	83	73	27	75	110	73
> 40	11	10	2	6	13	9
*Total*	*114*	*100*	*36*	*100*	*150*	*100*
Year of EEF						
Experimental (2000–2010/11)	17	15	0	0	17	11
Clinical Approval (2011/12–2016)	97	85	36	100	133	89
*Total*	*114*	*100*	*36*	*100*	*150*	*100*
No. EEF Cycles						
1	65	57	21	58	86	57
2	35	31	11	30	46	31
3	10	9	1	3	11	8
< 3	4	3	1	3	5	3
Unrevealed^a^	0	0	2	6	2	1
*Total*	*114*	*100*	*36*	*100*	*150*	*100*
Total No. Eggs Stored						
< 5	7	6	7	19	14	9
5–10	25	22	11	31	36	24
11–15	20	17	5	14	25	17
16–20	25	22	6	17	31	21
21–25	18	16	2	6	20	14
26–30	7	6	1	3	8	5
31–35	6	5	2	5	8	5
36–40	3	3	0	0	3	2
> 40	3	3	0	0	3	2
Unrevealed^a^	0	0	2	5	2	1
*Total*	*114*	*100*	*36*	*100*	*150*	*100*

^a^For religious and cultural reasons

More than half of the women (57%) undertook only one EEF cycle, and one-third (31%) undertook two cycles. These figures did not vary significantly between the two countries, although slightly more women in the US (12%) than in Israel (6%), undertook a third or higher-order cycle. On average, nearly 18 eggs per woman were retrieved and frozen among the US group, versus 13 in Israel.

As shown in Table 2, women who froze their eggs in both countries were highly educated. Only four women had *not* graduated from college due to successful careers in the performing arts or military. The rest of the women had considerable educational achievements. One-quarter of the women had completed bachelors’ degrees (25%), and the rest had earned advanced degrees, including master’s degrees (43%), medical degrees (15%), doctoral degrees (8%), and law degrees (5%). More than 10% of the American women had dual graduate degrees (e.g., MD-PhD, MD-MPH, MPP-PhD). More than half (58%) had attended Ivy League universities or other “elite” US academic institutions.

**Table 2:** Educational Achievement and Relationship Status of Women Undertaking Elective Egg Freezing (EEF) in the US and Israel

	US		ISRAEL		TOTAL	
	*n*	%	*n*	%	*n*	%
HIGHEST DEGREE						
High School	0	0	1	3	1	1
Associates Degree (2-Year)	1	1	0	0	1	1
Professional Arts Performance	2	2	0	0	2	1
Bachelors	23	20	14	39	37	25
Masters	52	45	13	36	65	43
MD	16	14	7	19	23	15
PhD	11	10	1	3	12	8
JD	8	7	0	0	8	5
MD-PhD	1	1	0	0	1	1
Total N	114	100	36	100	150	100
RELATIONSHIP STATUS AT TIME OF EEF						
Single						
Being Single	59	51	25	70	84	56
Divorced or Divorcing	19	17	6	17	25	17
Broken Up	16	14	2	5	18	12
*TOTAL SINGLE*	*94*	*82*	*33*	*91*	*127*	*85*
Partnered (Unstable)						
Relationship Too New or Uncertain	6	5	1	3	7	5
Partner Refuses to Have Children	2	2	0	0	2	1
Partner Has Multiple Partners	2	2	0	0	2	1
*TOTAL UNSTABLE PARTNERSHIPS*	*10*	*9*	*1*	*3*	*11*	*7*
Partnered (Stable)						
Not Ready to Have Children	10	9	2	5	12	8
*TOTAL STABLE PARTNERSHIPS*	*10*	*9*	*2*	*0*	*12*	*8*
Total N	114	100	36	100	150	100

Given these high levels of education, both the American and Israeli women in this study were professional women, whose careers were devoted to, among others, the health fields, basic and applied sciences, government and law, diplomacy and foreign service, academia, business management, information and technology, entrepreneurship, media and communications, human resources, the arts, the military, and beyond.

As shown in Table 2, 85% of these highly educated professional women were single at the time of EEF, either because they had no partner, were divorced, or had recently broken up from long-term relationships. Only one of these women had born a child from a prior relationship, which had ended. All of the other unpartnered women were childless at the time of EEF. Among the 15% of women who were partnered at the time of EEF, half of these relationships were unstable for the reasons outlined in Table 2. Only 10 (7%) of the women in this study, all of whom were American, were stably partnered at the time of EEF with men who hoped to have children with them in the future, even though most of the men were “not ready” yet.

Table 3 describes the post-EEF life circumstances of women at the time of our interviews. More than three-quarters of women (78%) were still single, while 22% were partnered (with either the same or a new partner). Seven percent of the partnered women had gone onto marry. However, as shown in Table 3, there were often significant differences in age, education, and reproductive history among women and their partners (e.g., a 38-year-old woman with a 55-year-old divorced man with children, or a woman ER physician with a high-school-educated paramedic). Only 7% of women described themselves as being in “equal” partnerships in terms of their partners’ education, age, and reproductive history (i.e., no children from a prior relationship).

**Table 3:** Relationship Status and Reproductive Outcomes Following Elective Egg Freezing (EEF) among Study Participants in the US and Israel

	US		ISRAEL		TOTAL	
	*n*	%	*n*	%	*n*	%
Years Elapsed Since EEF Undertaken						
Same year	40	35	1	3	41	27
1 year	28	25	12	33	40	26
2 years	21	18	13	36	34	23
3 years	12	11	6	17	18	12
4 years	7	6	3	8	10	7
5 or more years (5–11)	6	5	0	0	6	4
Wouldn’t reveal date of EEF	0	0	1	3	1	< 1
*Total*	*114*	*100*	*36*	*100*	*150*	*100*
Relationship Status Following EEF (@ Time of Interview)						
Still Single	89	78	27	75	116	78
Partnered	17	15	6	17	23	15
Married	8	7	3	8	11	7
*TOTAL*	*114*	*100*	*36*	*100*	*150*	*100*
Status of Those Women Partnered/Married						
Equal Partnership (Education, Age, No Children from Prior Relationship)	6	5	5	14	11	7
Partner Divorced without Children	1	1	0	0	1	1
Partner Divorced with Children	7	6	2	5	9	6
Partner Significantly Younger	3	3	1	3	4	3
Partner Significantly Older/Retired	2	2	1	3	3	2
Partner Significantly Less Educated	1	1	0	0	1	.5
Partner Significantly Less Educated/Divorced	1	1	0	0	1	**.**5
Partner Significantly Less Educated/Divorced with Children	1	1	0	0	1	.5
Partner Significantly Less Educated/Younger	1	1	0	0	1	.5
Partner with Alcohol or Legal Issues	2	2	0	0	2	1
*TOTAL AND PERCENT OF TOTAL N*	*25*	*22*	*9*	*25*	*34*	*22*
Pregnancy and Live Birth Outcomes Post EEF (@ Time of Interview)						
Child Born from Frozen Oocyte Conception	1	1	0	0	1	.5
Child Born from Natural Conception (No Frozen Oocytes Used)	3	2	1	3	4	3
Child Born from Donor Sperm (Single Mother by Choice, No Frozen Oocytes Used)	2	2	1	3	3	2
Child Born from IUI, IVF or Surrogacy (No Donor Sperm, No Frozen Oocytes Used)	4	3	0	0	4	3
Currently Pregnant from Frozen Oocyte	1	1	0	0	1	.5
Currently Pregnant from Natural Conception	2	2	1	3	3	2
Currently Pregnant from Donor Sperm (Single Mother by Choice, No Frozen Oocytes Used)	0	0	2	5	2	1
*TOTAL AND PERCENT OF TOTAL N*	*13*	*11*	*5*	*14*	*18*	*12*
Women Who Had Used Frozen Oocytes (by Time of Interview)						
All Oocytes Thawed, One Live Birth, One Blastocyst Remaining	1	1	0	0	1	.5
All Oocytes Thawed, Currently Pregnant, 24 Embryos Remaining	1	1	0	0	1	.5
All Oocytes Thawed, No Fertilization	8	7	0	0	8	5
*TOTAL AND PERCENT OF TOTAL N*	*10*	*9*	*0*	*0*	*10*	*6*

As also shown in Table 3, some women, whether partnered or not, decided to have children, with or without their frozen eggs. Twelve women in the study (10 American, 2 Israeli) had born children and six were currently pregnant at the time of the interviews. Few of these women had relied on their frozen eggs to become pregnant. Only 10 (6%) of the 150 women interviewed, all of them American, had pursued reproduction using frozen oocytes. Eight women had thawed all their eggs in an attempt to become pregnant, but only one had delivered a child and one had learned that she was pregnant at the time of the interview. The overall usage of frozen eggs remained low, as did the rate of frozen-egg conceptions.

### Qualitative findings

The socio-demographic findings above suggest that the lack of a stable partnership is the primary motivation among highly educated professional women in both the US and Israel. This result is supported by qualitative findings from women’s interviews. Women in the study routinely explained that they had been unable to find a stable, committed relationship with a man who also wanted to have children with them. Women often explained that throughout their educational and career-building years, they had attempted to find a compatible male partner, with whom they could build a family. When they had been unable to find one, they had pursued EEF, usually in their mid- to late 30s, but sometimes in their early 40s, in an effort to preserve their remaining reproductive potential.

In fact, in our study of 150 American and Israeli women, only one woman—who was American, and at age 30, the second youngest woman in the study—told us that she had explicitly used EEF to postpone her fertility “en route” to becoming a successful entrepreneur. Another American woman, age 33, was happy to have finally passed the difficult Foreign Service exam; she froze her eggs before her deployment to Latin America, where her career was just taking off. With the exception of these two women, the rest of the women in this study, both American and Israeli, did not pursue EEF for career-related purposes—as a means of postponing childbearing on the way to a better job or professional advancement. Rather, EEF was being used late in women’s reproductive lives because of ongoing reproductive partnership obstacles.

During interviews, both American and Israeli women often volunteered their insights—and considerable frustrations—over having to defer motherhood due to the lack of a partner. Women offered a variety of experiential perspectives on the lack of a partner problem in their own lives and for educated women more generally. Women’s assessments could be summarized and categorized in four main ways:

#### Women’s higher expectations

Women in this study addressed generational changes in women’s expectations for egalitarian partnerships. Women described how their parents, especially mothers, had encouraged them to “have it all,” and thus they had been raised to believe in gender equality and egalitarian relationships at home and at work. Thus, they hoped not to “settle” for a man who was less educated, less professionally accomplished, or less committed to similar interests and life goals. Many women in both the US and Israel said that they were still hoping to find the “right” person—the “soulmate” they were “meant” to be with. Searching for this person took time and commitment, but could prevent the fearful outcome of “settling for less” or entering into a “bad marriage.”

#### Men’s lower commitments

Having said this, women in the study, especially in the US, were skeptical about men of their generation, and whether these men shared the same desires and life goals. Women pointed out that men were not necessarily socialized in the same way to want egalitarian relationships with professional women, with whom they could balance the burdens and responsibilities of family life. Women in this study described men’s increasing “commitment phobia,” particularly men who were the “children of divorce” and were not sanguine about the virtues of either marriage or fatherhood. Furthermore, women in the US portion of the study, particularly on the West coast, described the “Peter Pan” syndrome—i.e., boys (in men’s bodies) who never grow up. These men were often described as not able to hold steady jobs, sometimes living with their parents (or being subsidized by them), and unable to fulfill the roles assumed by adult men in society. Furthermore, in the San Francisco Bay Area and other “progressive” cities, women described the growing phenomenon of “polyamory”—namely, millennial-generation men’s desires to have multiple, open relationships with “primary” and “secondary” female partners. In short, women in this study described men’s lowered commitments to fidelity, marriage, and parenthood—the trifecta often expected within traditional, heteronormative family structures.

#### Skewed gender demography

Beyond changing gender expectations on the part of both women and men, there was clear acknowledgment by many of the women in both countries that men of similar backgrounds—namely, single, college-educated, professionals, often with advanced degrees and high earnings—were simply hard to find. As one woman explained it succinctly, “the caliber of women is just higher than the caliber of guys.” This lament was especially true among American women on the East Coast, and particularly in New York City and Washington, DC, metropolitan areas that are known (via media reports) to have higher percentages of educated women than men. Women in those cities often lamented the dearth of “available” (and heterosexual) male partners in the skewed gender landscapes in which they were living. Furthermore, women often described their difficulties in “dating down” to less educated or less successful men. They characterized such relationships as fraught with “intimidation” on the part of men, who were generally emasculated by a woman’s superior professional status, living situation, or earnings.

Furthermore, because women in many societies, including the US and Israel, have traditionally been told to marry “up” (hypergamy) while men marry “down” (hypogamy) in terms of age, class, education, salary, and so on, trying to reverse this entrenched gender norm was inherently problematic on many levels, according to most women in the study. For one, they pointed out that most men were “ageist”—very reluctant to marry an older woman, especially one in her late 30s or early 40s who might place “pressure” on a partner to have children immediately.

#### Self-blame

Women who found themselves in this position—without partners in their mid- to late-30s—sometimes posed the “Why me?” question out loud in their interviews. Often with sadness, women expressed their amazement and disbelief that they had somehow “ended up” without a partner. Yet, they often added that they knew (many) other professional women in this situation. In both countries, but particularly in Israel, women sometimes blamed themselves for not finding a partner, criticizing themselves for being too “picky,” only attracted to “alpha males,” or that they had let a “good one” get away. Some women surmised that they were not attractive enough to men, or had not put enough “energy” into dating (especially online dating, which was widespread in our study population).

The inability to find a partner was a source of both frustration and anguish among women in both countries. As one American woman, an academic physician in her mid-30s, put it, “If I found a man, I’d move to Alaska! But most men don’t want relationships. They just want to meet and date. And most women won’t go out with the [uneducated] check-stand dude, but men will. So I think I have about a .09% chance of meeting someone. And meanwhile, I was feeling like, ‘OMG, my biological clock, it’s ticking, it’s ticking, it’s ticking,’ you know? So, even though I’m 1,000% happy I did it [EEF], it felt somewhat like a defeat. I felt like I gave up, because I couldn’t find a man.”

## Discussion

To date, this is the largest interview-based study of women who have undertaken EEF, and the only one with an international comparison. As a binational study, the research was designed to assess both similarities and differences in American and Israeli women’s: 1) life circumstances, 2) socio-demographic characteristics, and 3) motivations for undertaking EEF.

In all three realms, striking similarities were found across the two groups of women. In both countries, “lack of a partner” was the primary and driving force behind healthy women’s decisions to undergo EEF as a fertility preservation option. This dearth of male partners among the highly educated professional women in this study held true across both countries. It was a finding that was clearly expressed by women themselves as they reflected on their own EEF motivations and experiences.

Given this situation, an important question remains open for discussion: Namely, where are the “missing men,” who should presumably want to couple with these accomplished women? Should lack of a partner be taken for granted as a “natural fact” of educated women’s reproductive lives?

Here, we point to an underlying cause of this “man deficit,” one that has been carefully described and analyzed by one of us, Jon Birger [23], with particular focus on the US. Using US census data, Birger shows that there are 5.5 million university-educated women in their 20s (ages 22–29) in the US for only 4.1 million university-educated men. This is a ratio of 4:3. Between the ages of 30 and 39—when women start freezing their eggs—there are 7.4 million university-educated American women for only 6 million university-educated American men. This is a ratio of 5:4. Adding the two groups together, there are nearly 3 million more university-educated women than university-educated men in women’s prime reproductive years in America. To quote Birger ([23], p.3), “These lopsided gender ratios may add up to a sexual nirvana for heterosexual men, but for heterosexual women—especially those who put a high priority on getting married and having children in wedlock—they represent a demographic time bomb.”

What Birger calls a “massive undersupply” of university-educated men in America: 1) is growing over time as young women enter universities at much higher rates; 2) has reached a new high of 37% more American women than men in higher education, according to the most recent census data; 3) makes the long-term prospects for millennial-generation women decidedly worse; and 4) is particularly acute in major US cities such as Washington, DC, New York, and Miami, where university-educated women tend to cluster, but now outnumber university-educated men by the hundreds of thousands [23].

Beyond the US, this “man deficit” appears to be emerging around the world. As seen in Table 4, the most recent World Bank data from 2012 to 2016 show that women significantly outstrip men in higher education in at least 70 countries where data are available [24]. For example, this includes Australia, with 41% more women than men in higher education, as well as many other high-income countries including Belgium, France, Italy, New Zealand, Norway, Sweden, and the United Kingdom. These educational disparities are also emerging in many non-Western countries, including Argentina, China, Cuba, Lebanon, Malaysia, Panama, South Africa, Thailand, and Tunisia, to name only a few.

**Table 4:** Countries Where Women Significantly Outnumber Men in Higher Education^a^

No.	Country	F/M Ratio	% More Women than Men in Higher Education
1.	Albania	1.39865994	40%
2.	Algeria	1.55961001	56%
3.	Argentina	1.61947	62%
4.	Armenia	1.12863004	13%
5.	Aruba	2.26302004	126%
6.	Australia	1.40989006	41%
7.	Austria	1.20158994	20%
8.	Bahrain	1.92068005	92%
9.	Belarus	1.32676005	33%
10.	Belgium	1.31350005	31%
11.	Belize	1.60633004	61%
12.	Bermuda	2.31813002	132%
13.	Botswana	1.43773997	44%
14.	Brazil	1.39809	40%
15.	Canada^b^	1.29524887	30%
16.	Chile	1.13678002	14%
17.	China	1.18620002	19%
18.	Colombia	1.16246998	16%
19.	Costa Rica	1.30727994	31%
20.	Croatia	1.35680997	36%
21.	Cuba	1.42532003	43%
22.	Czech Republic	1.40742004	41%
23.	Estonia	1.53139997	53%
24.	Finland	1.20589006	21%
25.	France	1.22571003	23%
26.	Georgia	1.21904004	22%
27.	Guyana	2.03288007	103%
28.	Hong Kong SAR, China	1.16025996	16%
29.	Hungary	1.25191998	25%
30.	Iceland	1.71160996	71%
31.	Indonesia	1.1243	12%
32.	Ireland	1.09338999	9%
33.	Israel	1.3829	38%
34.	Italy	1.35718	36%
35.	Jamaica	1.72571003	73%
36.	Jordan	1.11230004	11%
37.	Kazakhstan	1.23714995	24%
38.	Kuwait	1.61944997	62%
39.	Latvia	1.42805004	43%
40.	Lebanon	1.15689003	16%
41.	Lithuania	1.46904004	47%
42.	Luxembourg	1.13515997	14%
43.	Macao SAR, China	1.32536995	33%
44.	Macedonia, FYR	1.24822998	25%
45.	Malaysia	1.52705002	53%
46.	Malta	1.37038004	37%
47.	Mongolia	1.38279998	38%
48.	Myanmar	1.22817004	23%
49.	Netherlands	1.10478997	10%
50.	New Zealand	1.35090995	35%
51.	Norway	1.45779002	46%
52.	Palau	1.54859996	55%
53.	Panama	1.49242997	49%
54.	Philippines	1.28163004	28%
55.	Poland	1.52178001	52%
56.	Portugal	1.13217998	13%
57.	Puerto Rico	1.40998995	41%
58.	Romania	1.23240995	23%
59.	Russian Federation	1.21165001	21%
60.	Serbia	1.33327997	33%
61.	Slovak Republic	1.54595995	55%
62.	Slovenia	1.44420004	44%
63.	South Africa	1.48450994	48%
64.	Spain	1.17773998	18%
65.	Sri Lanka	1.53942001	54%
66.	St. Lucia	1.90204	90%
67.	Sweden	1.52547002	53%
68.	Syrian Arab Republic	1.13739002	14%
69.	Thailand	1.41378999	41%
70.	Tunisia	1.65129006	65%
71.	Ukraine	1.15558004	16%
72.	United Kingdom	1.30744004	31%
73.	United States	1.36754	37%

^a^Based on the most recent World Bank data available from 2012 to 2016, as collected by the United Nations Educational, Scientific, and Cultural Organization (UNESCO) Institute for Statistics. https://data.worldbank.org/indicator/SE.ENR.TERT.FM.ZS?end=2011&name_desc=false&start=1970

^b^Statistics Canada. https://www150.statcan.gc.ca/n1/en/type/data#tables

In Israel, where part of our study was conducted, women have surpassed men at all academic levels. Between the years 1970–2013, the percent of female master’s students soared from 26 to 61%, and at the doctoral level, from 19 to 52% [25]. In the 2010–2011 academic year, women comprised 57% of undergraduates, 60% of master’s students, and 52% of PhD students [26]. In the academic year 2015–2016, women undergraduate students outnumbered men by 21% (and 40%, if teachers’ colleges are included). At the master’s level, the gap reached 62.5% [26]. Although women are still underrepresented in fields like computer sciences, their percentage has increased dramatically in prestigious, well-remunerated fields, including medicine, engineering, architecture, physics, and law. In fact, women now make up the majority of medical and law students in Israel [27].

As shown in our study, these educational disparities are clearly “creeping into” educated women’s lives. Table 3 reveals that women who partner may make “unequal” alliances with older, younger, or divorced men, including men with children from previous relationships, and often with significantly less education. As noted earlier, this phenomenon is known in anthropological terms as hypogamy—when a person marries “down.” Traditionally, women in societies around the world have tried to achieve hypergamy, or marrying “up,” in an attempt to secure a better future for themselves and their children. However, as women rise educationally around the globe, and men no longer keep pace, these trends may be reversing.

In a recent report entitled “Wayward Sons: The Emerging Gender Gap in Labor Markets and Education,” the authors conclude that “Although a significant minority of males continues to reach the highest echelons of achievement in education and labor markets, the median male is moving in the opposite direction” [28]. Overall, less-educated men in the US and in many other countries around the world are losing ground, not only in labor markets, but in marriage markets and parenthood, as reported by this and other studies [29].

What we see, then, are the difficult choices currently facing educated women in the US, Israel, and in many other nations regarding partnership and family formation [30, 31]. In this new era of EEF, “to freeze or not to freeze” has become the leading question among societies’ most educated women [13]. Indeed, EEF is not about women who are intentionally “delaying” childbearing or “postponing” their fertility in order to “lean in” to their careers [32]. Rather, EEF appears to be a technological concession among highly educated professional women who are grappling with gender-based socio-demographic disparities well beyond their individual reproductive control. Yet, given that knowledge of the educational gender gap is not widespread, women may end up engaging in self-blame—a negative discourse that author Sarah Eckel has questioned in her book *It’s Not You: The 27 (Wrong) Reasons You’re Single* [33].

To date, very few in-depth studies of this highly educated, egg-freezing population have been conducted. This study is by far the largest (e.g., 150 women interviewed versus 31 in the study by Baldwin [19, 20] and Baldwin et al. [18, 21]). However, as a large, binational, qualitative study, there were some inherent design and methodological limitations that must be acknowledged in assessing the aforementioned results. First, the overall number of participants recruited in the two countries was unequal, reflecting national differences in population size and, hence, EEF uptake. Second, the American and Israeli women participating in this study were recruited from a small number of metropolitan areas, thereby limiting the generalizability of the findings. Third, in the two countries, women were recruited into the study somewhat differently, and interviewed by two different medical anthropologists in two different languages, perhaps shaping their responses in unexpected ways. Furthermore, the numbers of women who were contacted in each clinic and each country varied considerably. Thus, women who chose not to participate in the study may have differed significantly from those women who volunteered; but the rates of non-response or lack of follow-up could not be calculated because of the less controlled, qualitative study design. All of these are sources of potential bias that could not be avoided.

## Conclusion

The major finding to emerge from this binational study was the “lack of partner” problem in highly educated professional women’s lives, leading them to EEF by their late 30s. A remarkable degree of consistency emerged in the findings from the two countries, as shown in Tables 1, 2, and 3. Furthermore, for a qualitative study of this nature, the sample size was quite large, and the in-depth nature of women’s interviews served to increase the validity of the study findings.

Instead of achieving motherhood, this cohort of exceptional, “30-something” women found themselves in what author Melanie Notkin ([34], p. xxi) has called “otherhood”—“single and approaching the end of our fertility.” Such otherhood has led to what Notkin calls “circumstantial infertility”—women waiting for love, not wanting to settle for a lesser love, but also realizing with trepidation that a loving partnership may not arrive in time for motherhood. Thus, these women’s childlessness is undesired and circumstantial, rather than the result of intentional fertility “postponement” and reproductive “choice.”

Given the lack of partner problem that is leading otherwise healthy women to pursue EEF, we conclude with two recommendations that seem to emerge from this study. First, it is important for IVF clinicians to become aware of, and sensitive, to the overarching lack of a partner problem facing their EEF patients. EEF patients may need different forms of social and emotional support, as well as patient-centered care, when they enter the couples-oriented world of IVF. Second, this study speaks to the need for additional qualitative research with women themselves as they both consider and undertake EEF in diverse national settings. We need to understand why some women who seek information about EEF decide against it, and why others decide to move forward, like the American and Israeli women who participated in this study. Future global empirical research of this nature will serve to facilitate worldwide comparisons of the underlying socio-demographic forces and gender-based disparities leading to the burgeoning uptake of EEF among otherwise healthy women around the globe.

## Abbreviations

EEF: Elective egg freezing
IVF: In vitro fertilization
